# Method for Assessing the Motor Coordination of Runners Based on the Analysis of Multichannel EMGs

**DOI:** 10.1155/2023/7126696

**Published:** 2023-05-19

**Authors:** Ren Sun, Shuijun Su, Quantao He

**Affiliations:** ^1^Department of Physical, Beijing Institute of Technology, Zhuhai 519000, Guangdong, China; ^2^José Rizal University, Mandaluyong City 1552, Metro Manila, Philippines; ^3^Sport School of Shenzhen University, Shenzhen 518000, Guangdong, China

## Abstract

In this paper, we propose a method to evaluate the motor coordination of runners based on the analysis of amplitude and spatiotemporal dynamics of multichannel electromyography. A new diagnostic index for the coordination of runners was proposed, including the amplitude of electromyography, the spatiotemporal stability coefficient, and the symmetry coefficient of muscle force. The motor coordination of 13 professional runners was studied. Detailed anthropometric information was recorded about the professional runners. It has been found that professional athletes are characterized by the stability of movement repetition (more than 83%) and the high degree of symmetry of muscle efforts of the left and right legs (more than 81%) regardless of the changes in load during running at a speed of 8–12 km/hr. Scientific and technological means can support the scientific training of athletes. The end of the Winter Olympic Games has shown us the powerful power of a series of intelligent scientific equipment, including electro-magnetic gun, in sports training. We also look forward to the continuous innovation of these advanced technologies, which will contribute to the intelligent development of sports scientific research.

## 1. Introduction

Coordination motor abilities (CMA) are important qualitative and quantitative characteristics of athletes' motor activity. The CMA can reflect the level of physical fitness and the degree to which an athlete has mastered new moves. At the same time, the CMA assesses the ability of athletes to change their athletic activities in unexpected situations [1]. The development of CMA is a background for sport-specific techno-tactical mastery [2, 3]. High levels of CMA improve the quality and speed of mastering new motor functions. Some aspects of CMA have recently been developed. These are as follows: the concept and variation of their training, the structure of the CMA of athletes in different sports, and the place of general and special coordination preparation in the long-term training system [2], which is good for the mastery of sports-specific tactics.

An analysis of recent studies shows that nowadays, pedagogical testing using special exercises (for instance, The Child Physical Coordination Assessment Tool (KTK-test) or Standard Motor Coordination Assessment Test for Children (MABC-2)) [4] and expert assessment [4–6] are the most popular methods used for investigating CMA in sports. At the same time, in medicine, methods of kinematic metering [7–10] and electrophysiological methods [11–14] are primarily used to study motor coordination. Electrophysiological and kinematic methods are mainly used in laboratory studies. Complex and expensive systems, trained and qualified personnel, and special methods of analyzing data are all important features of laboratory research. In addition, the movements themselves are of complex structural and functional composition. For this reason, new algorithms for processing biomechanical and electrophysiological data shall be developed [15].

This scientific paper suggests the use of multichannel electro-magnetic gun (EMG) for the analysis of the motor coordination of runners. Such an approach makes it possible to assess the degree and the nature of the consistency and proportionality of muscles involved in performing controlled motor action [16]. To expand the diagnostic potential of EMG signals, a combined analysis using spatiotemporal (motion) motion parameters is proposed. A number of scientific works show the effectiveness of EMG when used specifically for research in the field of sports [17, 18], including investigations of motor coordination [19]. With the continuous development of technology, surface EMG has been widely used in biomechanics, sports rehabilitation, human–computer interaction, and other fields. Surface electromyography in sports science research mainly focuses on muscle strength and muscle fatigue research. Now, surface electromyography has been widely used in muscle reaction time research, functional analysis of muscle activity, muscle coordination when completing a movement, muscle fiber composition analysis, muscle contraction speed research, muscle stability, coordination research, and so on [20].

The application of surface electromyography in sports scientific research [21, 22] is of great significance. It is helpful to establish a scientific training method by detecting the surface electromyographic signals of athletes and reflecting the fatigue [23] and excitation state of muscles in time. It is embodied in the following aspects: biomechanics and risk factors analysis of runners [24–29], using surface electromyography to measure nerve conduction velocity, using surface electromyography to assess nerve and muscle function, using surface electromyography to evaluate muscle strength, using surface electromyography to analyze movement [30–33].

It is important to note that there is a lot of work related to electromyography that aims to study pathological conditions or individual muscles in the field of running. However, EMG-related work to study the entire movement has not been reported [34–38]. In addition, there is scientific literature on the construction of muscle activity patterns and muscle synergies [39, 40]. Some papers focus on quantifying changes in muscle activity and coordination, such as speed, duration, mechanical restraint, etc. [41–44]. Sports science focuses on the study of human movement, which is mainly powered by muscles, so it is natural to use electromyography. For decades, sports workers at home and abroad have done a lot of work in opening up new fields of application of EMG in sports scientific research. At present, most studies devoted to the biomechanical analysis of running are only exploratory. Published papers have only studied the formation and change mechanisms of movement patterns under the influence of external and internal factors. Surface electromyography is the bioelectrical signal of muscle activity guided, recorded, and collected by surface electrodes when skeletal muscle is excited. EMG mostly uses a single electrode or double electrode placed in the muscled abdomen to extract the electromyographic signal, but there is an uneven activation state in skeletal muscle during movement, and few electrode channels extract signals due to different electrode positions, so it is difficult to complete accurate muscle activation analysis and muscle force estimation and other tasks. In this paper, we creatively propose a method to evaluate the motor coordination of runners based on the amplitude and spatiotemporal dynamic analysis of multi-channel electromyography. This paper provides a fast and effective method to evaluate the motor coordination of runners, which provides feasible conditions for the practical application of the motor pattern mechanism and the research task in the field of clinical medicine. In this paper, a new diagnostic index of motor coordination in runners was developed, including the amplitude and spatiotemporal stability coefficient of electromyography and the symmetry coefficient of muscle force, which provide a reference for accurate muscle activation analysis and muscle force estimation. The multichannel EMG amplitude and spatiotemporal dynamic analysis method provided in this paper have the advantages of nontrauma detection, no need for medical professionals to handle, easier to be accepted by the subjects, and so on, so it has a broader research application prospect.

## 2. Methods

### 2.1. Participants and Procedure

Professional marathon and long-distance (over 5,000 and 10,000 m) runners took part in the research. They are winners of national championships (Open Championship of the Republic of Belarus and the Cup of Belarus) and participants of international competitions (II European Games in Minsk, the European Athletics Indoor Championship 2017–2019, the XXXII Summer Olympic Games, etc.). Thirteen athletes (nine males and four females, aged 22–33) volunteered to participate in the study. The physical indicators of 13 athletes are shown in Table 1. All athletes were examined by the sports doctor at the time of the research. According to the examination results, the athletes tested had no health problems or injuries and no excessive exercise or dysfunction. The essence of the study was initially explained to the test athletes, and a test demonstration was conducted. All the participants provided written informed consent to participate in the study. The experiments were designed in accordance with the Declaration of Helsinki and approved by the expert commission of the Belarusian National Technical University.

Treadmills are often used in biomechanical studies, clinical practice, and training [45, 46]. The experiment was carried out on a treadmill. Before testing, the athlete had a necessary warm-up procedure: running for 5 min at a comfortable speed for the athlete (the speed of the warm-up run was chosen by the athlete). Investigations were carried out using the test with a stepwise increase of load [47–49]. The load was increased by steps of 1 km/hr every 30 s, starting from the speed of 8 km/hr and up to 712 km/hr. The athlete stood on the treadmill, after which the treadmill was set in motion. The speed of the treadmill was gradually increased until it reached 8 km/hr. Then the athlete ran for some time at the set initial speed, after which the signal was given to start testing and recording data. The signal was given when the athlete was ready. The speed range chosen for research corresponds to low-intensity running [50]. Jogging speeds can vary between 7 and 9 km/hr, while speeds over 10 km/hr are true running. In addition, treadmill-based stress tests [51] show that 8 km/hr is the traditional and most appropriate initial running speed. The technique of running at different speed ranges stays mostly unchanged. As running distances lengthen, the pace at which you run clearly has a decisive effect on running performance and physiological perception [52–54]. Maintain a steady pace during running, not fast and slow pace; not only the race performance will be more stable, but also the runner's physiological response will be easier to maintain a stable condition.

The following muscle groups were chosen for the study: rectus femoris, the lateral and medial heads of the quadriceps (m. vastus lateralis, m. vastus medialis), tibialis anterior, biceps femoris, and the lateral and medial heads of the gastrocnemius muscle (m. gastrocnemii medialis, m. gastrocnemii lateralis). The choice of the muscles was based on their active involvement in running [55]. Multichannel interference electromyography was used to study muscle movement during running, which can be used to assess muscle participation in various sports. This method can also assess the overall level of arousal and the proportion of muscle activity in each period of exercise [16].

Spatiotemporal parameters of running were calculated based on the signals of inertial micro-electro-mechanical systems gyroscopes. Gyroscope signals are represented as the projections of the angular velocity vector onto the axes associated with the object. By this means, the kinematic characteristics of movements can be analyzed [56].

### 2.2. Instruments and Data Collection

The EMG signals of muscles and gyroscope were registered using the Trigno Wireless System with Trigno Avanti Sensors (Delsys Inc., USA). Each wireless sensor can record one EMG channel (bandwidths 10–850 Hz, input range 22 mV, resolution 16 bits) and three gyroscope channels (three coordinate axes, sampling rate of 741 sa/s, resolution 16 bits) and transmits the data via Bluetooth over distances of up to 40 m [57].

The fixation and location of the sensors on the muscles under study, their orientation relative to the motor fibers, and the quality of skin surface preparation for the registration of superficial EMGs were made in accordance with SENIAM recommendations [58]. The sensors were fixed on selected muscles of the left and right legs using a material with a high degree of adhesion. The sensors do not cause any discomfort during training because of their compact size and wireless data transmission.

The EMG and gyroscope signals were recorded using Delsys Acquisition Software (Delsys Inc., USA). For further data analysis within the MATLAB technical computing environment, special software was developed, which, in terms of received data format (multichannel EMG signals and gyroscope signals), fully matches Trigno Wireless System [59].

### 2.3. Data Analysis

Here, we propose a method for assessing the motor coordination of runners based on the analysis of the amplitude and spatiotemporal dynamics of multichannel EMGs. The method includes the following steps:

1. Determination of the phases of running based on gyroscope signals.
2. Preprocessing of EMG signals.
3. Calculation of the symmetry of muscular efforts of the left and right limbs.
4. Determination of the amplitude and spatiotemporal dynamics of EMG profiles of muscles.

#### 2.3.1. Determination of the Phases of Running Based on Gyroscope Signals

To isolate the phases of running, we selected gyroscope signals from the sensors located on the tibialis anterior muscles of the left and right legs. The use of these signals makes it possible to quantitatively describe the movements of the tibia around the anatomical mediolateral axis, which allows the reliable identification of cycles for walking and running [60–62].

At the initial stage, adaptive filtering of the gyroscope signals is performed using a sliding averaging filter [63]:

(1) $$\begin{array}{c} y\left[i\right]=\frac{1}{N}\sum_{j=0}^{N-1}x\left[i+j\right], \end{array}$$

where *N* is the width of the filter window, *x*[*i*+*j*] is the input signal and *y*[*i*] is the output signal.

This filter is considered optimal for reducing random noise while maintaining the sharpness of the signal edges in the time domain. The window width of the moving averaging filter is selected dynamically and depends on the period of motion. The period of motion is determined based on the extrema of the autocorrelation function of gyroscope signals [64].

The criterion for distinguishing the phases of running is the local maxima of the gyroscope signal, which corresponds to the changes in the direction of the movement of a body link. The local maximal peaks of the angular velocity signal correspond to the middle of the unsupported part of the running locomotion [65].

**Figure 1:**
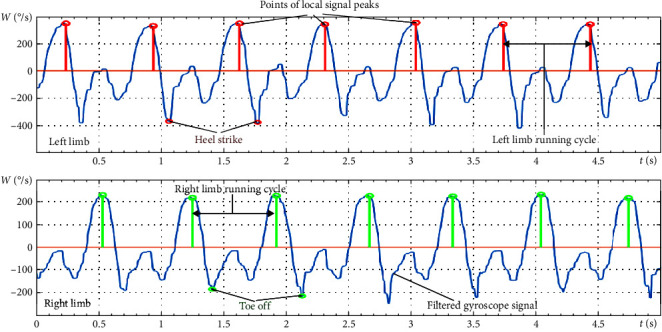
Isolation of running cycles.

Figure 1 shows an example of the selection of running cycles based on the local extrema of the angular velocity signals about the *x*-axis obtained from the gyroscope sensors located on the tibialis anterior of the left and right leg. Running speed in the presented example is 9 km/hr. The top graph illustrates the isolation of the running cycles for the left leg, and the bottom graph represents the right leg. In addition, the “heel strike” and “toe-off” points are indicated in the graphs.

#### 2.3.2. Preprocessing of EMG Signals

When analyzing biomedical signals, including EMGs, the reliability of the research results is determined by the quality of the recorded signals. Motor artifacts, high-frequency interference, mains frequency interference, and cross-talk from various biomedical significantly distort the useful EMG signals [63].

In this study, we digitally filter EMG signals to remove distortion in the spectrum of useful signals:

1. Butterworth high-pass filter with a cutoff frequency of 10 Hz is used to remove motion artifacts [63].
2. Butterworth notch filter with a notch frequency of 50 Hz is used to remove the main frequency [64].

#### 2.3.3. Calculation of the Symmetry of Muscular Efforts of the Left and Right Limbs

The formation of movements is often accompanied by different involvement of the right and left hemispheres in the control of a motor action resulting in the asymmetry of motor functions in the left and right limbs [66].

In order to obtain a quantitative assessment of muscle efforts during running, we calculated the energy of EMG signals in previously identified cycles of motor action in accordance with the author's methodology [67]. The EMG and gyroscope signals were recorded synchronously, so the running cycles for the left and right legs isolated on the basis of angular velocity were further used to divide the EMGs of the muscles by phases.

In general, the energy of a digital signal is viewed not as a physical quantity but as a tool for comparing different signals [64]. In the case of EMG, the energy of the signal is proportional to the force exerted by the muscle during a movement [68]. To assess the symmetry of the muscular efforts of the left and right legs during running, a correlation analysis of the envelopes of EMG energy signals of the same muscles on opposite limbs was performed. The EMG energy envelopes were determined by the application of spline interpolation using local signal maxima [69].

The cross-correlation coefficient [64] of the envelopes of EMG energy signals was calculated by the formula:

(2) $$\begin{array}{c} r_{12}\left(j\right)=\frac{1}{N}\sum_{n=0}^{N-1}x_{1}\left(n\right)\times x_{2}\left(n+j\right), \end{array}$$

where *x*_1_(*n*) × *x*_2_(*n*) are the digitized envelopes of EMG energy signals, each containing *N* elements, and *j* is the delay by which signal *x*_2_(*n*) is shifted relative to signal *x*_1_(*n*).

**Figure 2:**
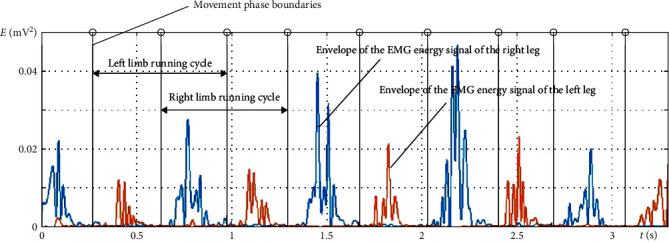
The envelopes of EMG energy signals are obtained from rectus femoris muscles of opposite limbs.

At the same time, for running, the delay *j* is equal to cycle/2 since the movements of the opposite limbs are performed in antiphase (Figure 2).

The symmetry coefficient of the muscular efforts of the left and right legs (*α*, measured in %) was calculated as the average value of the cross-correlation coefficients of the envelopes of EMG energy signals of all the muscles under study. This analysis makes it possible to reveal asymmetries in the motor function of the left and right limbs.

#### 2.3.4. Determination of the Amplitude and Spatiotemporal Dynamics of EMG Profiles of Muscles

In analyzing EMG, a pressing task is to identify the region with the greatest muscle activity (the positioning window of muscle activity). With distorted locomotion, the transformation of an EMG profile of the muscles frequently occurs. This changes the amplitude and duration of muscle electrical activity maxima or transfers the maxima to another phase of the motorcycle [70].

The muscular effort localization windows can be determined based on the isolation of the time interval with the maximum energy concentration of the EMG signal of the muscles in each phase of a movement. The isolation criterion for this time interval is the energy concentration of the EMG signal of the muscle, which accounts for at least 90% of the energy of the initial EMG signal during the exercise phase:

(3) $$\begin{array}{c} E_{f,T}=0.9\times E_{f}, \end{array}$$

where *E*_*f*,*T*_ is the energy of the muscle EMG signal within time interval *T* in phase *f*, *T* is the time interval of the localization window, *f* is the number of a movement phase, and *E*_*f*_ is the energy of the initial EMG signal of the muscle in phase *f*.

**Figure 3:**
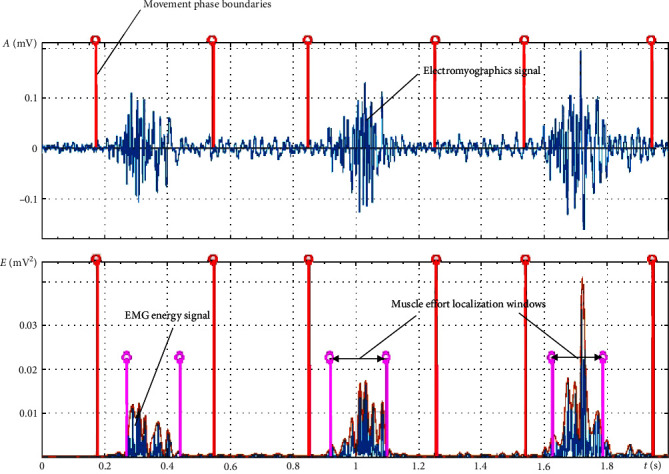
An example of isolation of the localization windows of muscular efforts in the running phases.

Figure 3 shows an example of isolation of the localization windows of rectus femoris muscular efforts of the right leg in the selected phases of running.

The dynamic of electrophysiological parameters during exercise is an indicator of the imperfection of exercise behavior. Because of increased muscle energy expenditure to correct and maintain motor behavior [71]. In this research, the assessment of the amplitude and spatiotemporal dynamics of muscle EMG profiles were carried out within 20 cycles of a movement. The following parameters were chosen for the analysis: the duration of the phases of movement *t*, the energy of the EMG signal in the localization windows *E*_*f*,*T*_^*i*^ and the time interval of the localization windows of muscle efforts *T*.

The coefficient of the stability of the EMG profiles of muscle is calculated in accordance with the following algorithm.

For each movement phase, the ratio of the duration of the muscle effort localization window to the phase duration Δ*t*^*i*^ is calculated for a repeated movement:

(4) $$\begin{array}{c} \Delta t_{f}^{i}=\frac{T_{f}^{i}}{t_{f}^{i}}, \end{array}$$

where *i* is the number of a movement cycle, *f* is the number of a movement phase, *T*_*f*_^*i*^ is the duration of the muscle effort localization window in the *f*th phase of the movement under investigation for the *i*th cycle, and *t*_*f*_^*i*^ is the duration of the *f*th phase of the movement under investigation for the *i*th cycle.

For each phase of movement, the average value of the EMG signal energy in the localization windows (*E*_*f*,*T*_^cp^) and the ratio of the duration of the muscle effort localization window to the phase duration (Δ*t*_*f*_^cp^) are calculated for a repeated movement:

(5) $$\begin{array}{c} E_{f,T}^{cp}=\frac{\sum_{i=1}^{N}E_{f,T}^{i}}{N}, \end{array}$$

where *N* is the number of movement cycles, and *E*_*f*,*T*_^*i*^ is the energy of the EMG signal in the localization window in the *f*th phase of the movement under investigation for the *i*th cycle.

(6) $$\begin{array}{c} \Delta t_{f}^{cp}=\frac{\sum_{i=1}^{N}\Delta t_{f}^{i}}{N}, \end{array}$$

where Δ*t*_*f*_^*i*^ is the ratio of the duration of the muscle effort localization window to the phase duration in the *f*th phase of the movement under investigation for the *i*th cycle.

For each phase of movement, the standard deviation of the EMG signal energy in the localization windows *σ*_*f*,*T*_^*E*^ and the ratio of the duration of the muscle effort localization window to the phase duration *σ*_*f*_^Δ*t*^ are calculated for a repeated movement:

(7) $$\begin{array}{c} \sigma_{f,T}^{E}=\sqrt{\frac{\sum_{i=1}^{N}{\left(E_{f,T}^{i}-E_{f,T}^{cp}\right)}^{2}}{N}}, \end{array}$$

(8) $$\begin{array}{c} \sigma_{f}^{\Delta t}=\sqrt{\frac{\sum_{i=1}^{N}{\left(\Delta t_{f}^{i}-\Delta t_{f}^{cp}\right)}^{2}}{N}}. \end{array}$$

The standard deviation of EMG signal energy in the positioning window and the ratio of the duration of the muscle effort positioning window to the phase duration represent the dynamic degree of the EMG parameters.

For each exercise stage, the stability coefficient of EMG signal energy in the positioning window and the stability coefficient of the ratio of muscle effort positioning window duration to phase duration was calculated:

(9) $$\begin{array}{c} k_{f,T}^{E}=\left(1-\frac{\sigma_{f,T}^{E}}{E_{f,T}^{cp}}\right)\times 100\%, \end{array}$$

(10) $$\begin{array}{c} k_{f}^{\Delta t}=\left(1-\frac{\sigma_{f}^{\Delta t}}{\Delta t_{f}^{cp}}\right)\times 100\%. \end{array}$$

The stability coefficient of the EMG signal energy in the localization windows and the stability coefficient of the ratio of the duration of the muscle effort localization window to the phase duration characterizes the degree of repeatability of EMG parameters of motor action from cycle to cycle.

Calculations of the integral coefficient of the amplitude and spatiotemporal stability of muscle EMG profiles.

The integral stability coefficient of muscle EMG profiles *K*_EMG_ is calculated as the average value of all the obtained stability coefficients of the EMG signal energy in the localization windows and the stability coefficients of the ratio of the duration of the muscle localization window of muscle efforts to the phase duration:

(11) $$\begin{array}{c} K_{EMG}=\sum_{m=1}^{M}\sum_{f=1}^{F}\frac{\left(k_{f,T}^{E}+k_{f}^{\Delta t}\right)}{2}/F\times M, \end{array}$$

where *F* is the number of movement phases, and *M* is the number of the muscles under study.

The coefficient of the amplitude and spatiotemporal stability of muscle EMG profiles lies in the range (0%–100%).

## 3. Results and Discussion

As a result of the research, the following parameters were calculated for each athlete: the integral coefficient of the amplitude and spatiotemporal stability of the EMG profiles of muscles (*K*_EMG_ (%)) for each load step (8, 9, 10, 11, and 12 km/hr) [72]. The coefficient reflects the degree of the repeatability of the EMG parameters of motor action from cycle to cycle.

The symmetry coefficient (*α*) of left and right leg muscles at each weight-bearing step, which can reveal the asymmetry of left and right limb motor function [73].

Table 2 demonstrates the data characterizing the individual dynamics of the muscle EMG profiles obtained during the test with a stepwise increasing load on the treadmill.

The analysis of the data obtained allows us to conclude that the stability of repetition of the movement for running speeds of 8–12 km/hr, regardless of the changes in load, is an inherent feature for recognized athletes. This feature evidences their highly developed motor coordination. At the same time, the maximum stability of the EMG profiles of athletes' muscles was observed at a load of 10 km/hr (*K*_EMG_ = 84.63 ± 1.76%), while the minimum stability was detected at a load of 11 km/hr (*K*_EMG_ = 82.97 ± 4.26%).

Table 3 demonstrates the data characterizing the individual symmetry of the muscle effort of opposite limbs obtained during the test with a stepwise increasing load on the treadmill. The table shows the averaged (mathematical expectation ± standard deviation) values of the symmetry coefficient of the muscle efforts of the left and right legs (*α* (%)) for all the load steps.

The analysis of the data obtained allows us to conclude that the stability of symmetry of the muscular efforts of the left and right legs for running speeds of 8–12 km/hr, regardless of the load, is an inherent feature for recognized athletes. This feature shows their highly developed motor coordination. At the same time, the following tendency is observed: the degree of symmetry of the action of the muscles of opposite limbs increases as the athlete's training experience increases (Pearson's correlation coefficient, *r* = 0.79).

## 4. Conclusions

Here, we propose a method for assessing the motor coordination of runners based on the analysis of the amplitude and spatiotemporal dynamics of multichannel EMGs. This method makes it possible to quantitative evaluation of running stability under various conditions as well as to reveal the asymmetry of the actions of paired muscle groups. A feature of the proposed method is that it reveals new diagnostic indicators of the motor coordination of runners: the coefficient of the amplitude and spatiotemporal stability of muscle EMG profiles and the symmetry coefficient of muscle efforts.

The studies carried out in this research showed the efficiency of the proposed approach in assessing the motor coordination of athletes. It has been found that recognized athletes are characterized by the stability of movement repetition (on average more than 83%) and the high degree of symmetry of muscle efforts in the left and right legs (on average more than 81%) regardless of the changes in load during running at a speed of 8–12 km/hr. These features evidence the athletes' highly developed motor coordination. In addition, a tendency for an increase in the symmetry degree of the action of the same muscles of opposite limbs with an increase in the level of an athlete's mastery (*r* = 0.79) has been revealed.

The source of the surface electromyographic signal is very complex; there are many interferences, and the signal is weak. Therefore, in the course of testing, the accuracy and reliability of the test must be put forward higher requirements. For example, various interference, such as power supply noise, electrode position, instrument accuracy, skin sweating degree, body posture, as well as the physiological state of the subject, may have a great impact on the test results. Surface electromyographic signals can only be obtained from superficial muscles, not from deep muscles, not to mention from a movement unit or a muscle fiber. Therefore, the surface electromyographic signal is affected by the number of motor units recruited, the degree of synchronization of motor units, the type of muscle fiber composition, subcutaneous fat thickness, and body temperature. In addition, there are many factors affecting the technical action, especially the technical action is the result of a series of continuous actions, with continuity. Surface electromyography is only a diagnostic means and can not be used as the sole criterion to judge the quality of motor movements.

The proposed method for assessing the motor coordination of runners based on the analysis of the amplitude and spatiotemporal dynamics of multichannel EMGs can be used to develop new criteria for evaluating the effectiveness of solving motoric tasks as well as for assessing the correctness of movement techniques and detecting critical errors that lead to injuries.

## Figures and Tables

**Table 1:** Detailed anthropometric information for professional runners.

S. no	Height (cm)	Weight (kg)	Gender information	Body mass index
1	169	62	Man	21.7
2	176	70	Man	22.6
3	164	49	Woman	18.2
4	175	68	Man	22.2
5	184	76	Man	22.4
6	181	73	Man	22.3
7	166	50	Woman	18.1
8	168	52	Woman	18.4
9	178	72	Man	22.7
10	170	54	Woman	18.7
11	182	74	Man	22.3
12	180	74	Man	22.8
13	177	71	Man	22.7

**Table 2:** Integrated stability coefficient of EMG (%) for athlete's workload at each step.

S. no	Stage 1 (8 km/hr)	Stage 2 (9 km/hr)	Stage 3 (10 km/hr)	Stage 4 (11 km/hr)	Stage 5 (12 km/hr)
1	85.05	79.20	84.24	86.75	80.63
2	83.16	83.19	83.63	82.68	85.77
3	86.40	86.67	86.52	87.79	87.15
4	86.13	85.57	85.31	84.66	82.77
5	84.46	81.22	85.91	82.81	82.65
6	82.61	80.17	84.06	77.74	82.46
7	85.96	84.51	85.37	86.32	85.37
8	85.65	87.42	84.39	85.67	81.25
9	84.71	87.97	86.59	79.67	87.10
10	70.80	87.84	82.03	72.91	86.13
11	80.22	87.06	82.17	87.17	86.31
12	84.82	84.94	82.46	82.68	84.53
13	80.12	82.07	87.49	81.70	85.20
Mean ± SD	83.09 ± 4.23	84.45 ± 3.02	84.63 ± 1.76	82.97 ± 4.26	84.41 ± 2.21

**Table 3:** Symmetry coefficient of muscular efforts of the left and right limbs.

Athlete	*α* (%)	Training experience
1	77.04 ± 7.97	Less than 10 years
2	70.44 ± 6.07	Less than 10 years
3	64.49 ± 5.13	Less than 10 years
4	79.53 ± 7.53	Less than 10 years
5	81.87 ± 4.32	Less than 10 years
6	72.56 ± 6.26	Less than 10 years
7	84.05 ± 6.97	From 10 to 15 years
8	83.29 ± 6.53	From 10 to 15 years
9	95.65 ± 3.12	Over 15 years
10	88.79 ± 4.28	Over 15 years
11	83.03 ± 3.64	Over 15 years
12	85.73 ± 4.95	Over 15 years
13	86.67 ± 3.00	Over 15 years

## Data Availability

The raw data supporting the conclusions of this article will be made available by the authors.

## References

[1] Sadowski J., Niźnikowski T. (2008). *Coordination Motor Abilities in Scientific Research*.

[2] Lyakh V., Sadowski J., Witkowski Z. (2011). Development of coordination motor abilities (CMA) in the system of long-term preparation of athletes. *Polish Journal of Sport and Tourism*.

[3] Issurin V. B., Lyakh V. I. (2017). Coordination abilities of athletes: basics of manifestation, evaluation and elucidation: a review. *Journal of Athletic Enhancement*.

[4] Olesen L. G., Kristensen P. L., Ried-Larsen M., Grøntved A., Froberg K. (2014). Physical activity and motor skills in children attending 43 preschools: a cross-sectional study. *BMC Pediatrics*.

[5] Jaakkola T., Watt A., Kalaja S. (2017). Differences in the motor coordination abilities among adolescent gymnasts, swimmers, and ice hockey players. *Human Movement*.

[6] Boichuk R., Iermakov S., Nosko M., Kovtsun V., Nosko Y. (2017). Influence of motor coordination indicators on efficiency of game activity of volleyball players at the stage of specialized basic training. *Journal of Physical Education and Sport*.

[7] Pomeshchikova I. P., Shevchenko O. O., Yermakova T. S. (2016). Influence of exercises and games with ball on coordination abilities of students with disorders of muscular skeletal apparatus. *Journal of Physical Education and Sport*.

[8] Sung P. S. (2014). A kinematic analysis for shoulder and pelvis coordination during axial trunk rotation in subjects with and without recurrent low back pain. *Gait & Posture*.

[9] Yi L. C., Sartor C. D., Souza F. T., Sacco I. C. N. (2016). Intralimb coordination patterns in absent, mild, and severe stages of diabetic neuropathy: looking beyond kinematic analysis of gait cycle. *PLOS ONE*.

[10] Cowley J. C., Gates D. H. (2017). Proximal and distal muscle fatigue differentially affect movement coordination. *PLOS ONE*.

[11] Yoo J. W., Lee D. R., Cha Y. J., You S. H. (2017). Augmented effects of EMG biofeedback interfaced with virtual reality on neuromuscular control and movement coordination during reaching in children with cerebral palsy. *NeuroRehabilitation*.

[12] Hesam-Shariati N., Trinh T., Thompson-Butel A. G., Shiner C. T., McNulty P. A. (2017). A longitudinal electromyography study of complex movements in poststroke therapy. 2: Changes in coordinated muscle activation. *Frontiers in Neurology*.

[13] Hawkes D. H., Alizadehkhaiyat O., Kemp G. J., Fisher A. C., Roebuck M. M., Frostick S. P. (2012). Shoulder muscle activation and coordination in patients with a massive rotator cuff tear: an electromyographic study. *Journal of Orthopaedic Research*.

[14] He J., Zhang D., Sheng X., Li S., Zhu X. (2015). Invariant surface EMG feature against varying contraction level for myoelectric control based on muscle coordination. *IEEE Journal of Biomedical and Health Informatics*.

[15] Davydova N. S. (2012). *Apparatno-Programmnyy Kompleks Mnogokanal’noy Elektromiografii Dlya Diagnostiki Dvigatel’nykh Navykov Cheloveka: [Hardware-Software Complex of Multichannel Electromyography for the Diagnosis of Human Motor Skills]*.

[16] Merletti R., Merletti D. (2016). *Surface Electromyography: Physiology, Engineering, and Applications*.

[17] Clarys J. P. (2000). Electromyography in sports and occupational settings: an update of its limits and possibilities. *Ergonomics*.

[18] Türker H., Sozen H., Turker H. (2013). Surface electromyography in sports and exercise. *Electrodiagnosis in New Frontiers of Clinical Research*.

[19] Hug F. (2011). Can muscle coordination be precisely studied by surface electromyography?. *Journal of Electromyography and Kinesiology*.

[20] Hawkes D. H., Khaiyat O. A., Howard A. J., Kemp G. J., Frostick S. P. (2019). Patterns of muscle coordination during dynamic glenohumeral joint elevation: an EMG study. *PLOS ONE*.

[21] Neal B. S., Barton C. J., Gallie R., O’Halloran P., Morrissey D. (2016). Runners with patellofemoral pain have altered biomechanics which targeted interventions can modify: a systematic review and meta-analysis. *Gait & Posture*.

[22] Becker J., Nakajima M., Wu W. F. W. (2018). Factors contributing to medial tibial stress syndrome in runners: a prospective study. *Medicine & Science in Sports & Exercise*.

[23] Brown A. M., Zifchock R. A., Hillstrom H. J., Song J., Tucker C. A. (2016). The effects of fatigue on lower extremity kinematics, kinetics and joint coupling in symptomatic female runners with iliotibial band syndrome. *Clinical Biomechanics*.

[24] Hein T., Janssen P., Wagner-Fritz U., Haupt G., Grau S. (2014). Prospective analysis of intrinsic and extrinsic risk factors on the development of Achilles tendon pain in runners. *Scandinavian Journal of Medicine & Science in Sports*.

[25] Mousavi S. H., Hijmans J. M., Rajabi R., Diercks R., Zwerver J., van der Worp H. (2019). Kinematic risk factors for lower limb tendinopathy in distance runners: a systematic review and meta-analysis. *Gait & Posture*.

[26] Mei Q., Fernandez J., Fu W., Feng N., Gu Y. (2015). A comparative biomechanical analysis of habitually unshod and shod runners based on a foot morphological difference. *Human Movement Science*.

[27] Willems T. M., Ley C., Goetghebeur E., Theisen D., Malisoux L. (2021). Motion control shoes reduce the risk of pronation-related pathologies in recreational runners: a secondary analysis of a randomized controlled trial. *Journal of Orthopaedic & Sports Physical Therapy*.

[28] Malisoux L., Chambon N., Delattre N., Gueguen N., Urhausen A., Theisen D. (2016). Injury risk in runners using standard or motion control shoes: a randomised controlled trial with participant and assessor blinding. *British Journal of Sports Medicine*.

[29] Jauhiainen S., Pohl A. J., Äyrämö S., Kauppi J.-P., Ferber R. (2020). A hierarchical cluster analysis to determine whether injured runners exhibit similar kinematic gait patterns. *Scandinavian Journal of Medicine & Science in Sports*.

[30] Floría P., Sánchez-Sixto A., Harrison A. J., Ferber R. (2019). The effect of running speed on joint coupling coordination and its variability in recreational runners. *Human Movement Science*.

[31] Aljohani M., Kipp K. (2020). Use of self-organizing maps to study sex- and speed-dependent changes in running biomechanics. *Human Movement Science*.

[32] Clermont C. A., Benson L. C., Bren Edwards W., Hettinga B. A., Ferber R. (2019). New considerations for wearable technology data: changes in running biomechanics during a marathon. *Journal of Applied Biomechanics*.

[33] Hollis C. R., Koldenhoven R. M., Resch J. E., Hertel J. (2021). Running biomechanics as measured by wearable sensors: effects of speed and surface. *Sports Biomechanics*.

[34] Morley J. J., Traum E. (2018). The effects of dorso-lumbar motion restriction on EMG activity of selected muscles during running. *Journal of Bodywork and Movement Therapies*.

[35] Esculier J.-F., Roy J.-S., Bouyer L. J. (2015). Lower limb control and strength in runners with and without patellofemoral pain syndrome. *Gait & Posture*.

[36] Baker R. L., Souza R. B., Rauh M. J., Fredericson M., Rosenthal M. D. (2018). Differences in knee and hip adduction and hip muscle activation in runners with and without iliotibial band syndrome. *PM&R*.

[37] Brown A. M., Zifchock R. A., Lenhoff M., Song J., Hillstrom H. J. (2019). Hip muscle response to a fatiguing run in females with iliotibial band syndrome. *Human Movement Science*.

[38] Garbalosa J. C., Elliott B., Feinn R., Wedge R. (2015). The effect of orthotics on intersegmental foot kinematics and the EMG activity of select lower leg muscles. *The Foot*.

[39] Cappellini G., Ivanenko Y. P., Poppele R. E., Lacquaniti F. (2006). Motor patterns in human walking and running. *Journal of Neurophysiology*.

[40] Santuz A., Ekizos A., Janshen L. (2018). Modular control of human movement during running: an open access data set. *Frontiers in Physiology*.

[41] Jewell C., Hamill J., von Tscharner V., Boyer K. A. (2019). Altered multi-muscle coordination patterns in habitual forefoot runners during a prolonged, exhaustive run. *European Journal of Sport Science*.

[42] Kyröläinen H., Avela J., Komi P. V. (2005). Changes in muscle activity with increasing running speed. *Journal of Sports Sciences*.

[43] Haudum A., Birklbauer J., Kröll J., Müller E. (2012). Constraint-led changes in internal variability in running. *Journal of Sports Science & Medicine*.

[44] Haudum A., Birklbauer J., Müller E., Birklbauer A. The influence of external perturbations on running kinematics and muscle activity before and after accommodation. *Journal of Sports Science & Medicine*.

[45] Souza R. B. (2016). An evidence-based videotaped running biomechanics analysis. *Physical Medicine and Rehabilitation Clinics of North America*.

[46] Milgrom C., Finestone A., Segev S., Olin C., Arndt T., Ekenman I. (2003). Are overground or treadmill runners more likely to sustain tibial stress fracture?. *British Journal of Sports Medicine*.

[47] Youqing S., Guodong X. (2011). *Recovery of Muscle Oxygen Content and Blood Lactic Acid after Incremental Load Exercise*.

[48] Xiaofeng Z., Qiong C. (2015). Experimental research on characteristics of myocardial repolarization in swimming athletes. *Journal of Shenyang Sport University*.

[49] Leqin C. (2011). Effects of increasing load exercise on cardiovascular function in adults with different BMI and exercise habits. *Journal of Beijing Sport University*.

[50] Nummela A., Keränen T., Mikkelsson L. O. (2007). Factors related to top running speed and economy. *International Journal of Sports Medicine*.

[51] Folland J. P., Allen S. J., Black M. I., Handsaker J. C., Forrester S. E. (2017). Running technique is an important component of running economy and performance. *Medicine & Science in Sports & Exercise*.

[52] Shunzheng W., Yuqiong L., Songfang W., Chensheng W., Zhaowei L. (2019). The average person’s marathon pace.

[53] Shunzheng W., Yuqiong L., Hesen W., Songfang W. (2018). How to pace a marathon.

[54] Shunzheng W., Yuqiong L., Yanlin H. (2014). The effect of Initial running pace on triathlon performance.

[55] Tortora G. J., Derrickson B. H. (2018). *Principles of Anatomy and Physiology*.

[56] Du Jaaa., Gerdtman C., Lindén M. (2018). Signal quality improvement algorithms for MEMS gyroscope-based human motion analysis systems: a systematic review. *Sensors*.

[57] DELSYS (2021). Trigno wireless system. https://delsys.com/trigno/research/.

[58] Hermens H. J., Freriks B., Merletti R. (1999). European recommendations for surface electromyography. *Roessingh Research and Development*.

[59] Davydova N. S., Vasiuk V. Е., Paramonova N. A., Mezhennaya М. М., Guseinov D. I. (2020). Algorithm for the analysis of kinematic characteristics of running. *Doklady BGUIR*.

[60] Greene B. R., McGrath D., O’Neill R., O’Donovan K. J., Burns A., Caulfield B. (2010). An adaptive gyroscope-based algorithm for temporal gait analysis. *Medical & Biological Engineering & Computing*.

[61] Aminian K., Najafi B., Büla C., Leyvraz P.-F., Robert P. (2002). Spatio-temporal parameters of gait measured by an ambulatory system using miniature gyroscopes. *Journal of Biomechanics*.

[62] Cutti A. G., Ferrari A., Garofalo P., Raggi M., Cappello A., Ferrari A. (2010). ‘Outwalk’: a protocol for clinical gait analysis based on inertial and magnetic sensors. *Medical & Biological Engineering & Computing*.

[63] De Luca C. J., Donald Gilmore L., Kuznetsov M., Roy S. H. (2010). Filtering the surface EMG signal: movement artifact and baseline noise contamination. *Journal of Biomechanics*.

[64] Akay M. (2012). *Biomedical Signal Processing*.

[65] McGrath D., Greene B. R., O’Donovan K. J., Caulfield B. (2012). Gyroscope-based assessment of temporal gait parameters during treadmill walking and running. *Sports Engineering*.

[66] Amunts K., Jäncke L., Mohlberg H., Steinmetz H., Zilles K. (2000). Interhemispheric asymmetry of the human motor cortex related to handedness and gender. *Neuropsychologia*.

[67] Davydova N., Davydov M., Osipov A., Mezhennaya M. (2019). Complex analysis of human movements based on the identification of amplitude-time characteristics of electromyographic patterns. *Global Journal of Research In Engineering*.

[68] Davydova N., Vasiuk V., Osipov A. (2019). Estimation of athlete coordination abilities based on the reproducibility analysis of the electromyographic patterns of complex coordination movements. *Journal of Engineering Science*.

[69] Unser M. (1999). Splines: a perfect fit for signal and image processing. *IEEE Signal Processing Magazine*.

[70] Davlet’yarova K. V., Korshunov S. D., Krivoshchekov S. G., Kapilevich L. V. (2020). Physiological parameters of motor adaptation in children with disability. *Human Physiology*.

[71] Davydova N. S., Mihuta I. Y., Osipov A. N., Boriskevich A. A., Vasyuk V. E., Hoholko A. A. (2012). Estimation of coordinating abilities of a person based on analysis of electromiographic patterns of movements. *Doklady BGUIR*.

[72] Yamane T., Yamasaki Y., Nakashima W., Morita M. (2023). Tri-axial accelerometer-based recognition of daily activities causing shortness of breath in COPD patients. *Physical Activity and Health*.

[73] Liu Q., Chen H., Song Y. (2022). Running velocity and longitudinal bending stiffness influence the asymmetry of kinematic variables of the lower limb joints. *Bioengineering*.

